# In search of noise-induced bimodality

**DOI:** 10.1186/1741-7007-10-89

**Published:** 2012-11-07

**Authors:** Kyung Hyuk Kim, Herbert M Sauro

**Affiliations:** 1 Department of Bioengineering, University of Washington, William H Foege Building, Box 355061, Seattle, WA 98195-5061, USA

## Abstract

Many biological studies are carried out on large populations of cells, often in order to obtain enough material to make measurements. However, we now know that noise is endemic in biological systems and this results in cell-to-cell variability in what appears to be a population of identical cells. Although often neglected, this noise can have a dramatic effect on system responses to environmental cues with significant and often counter-intuitive biological outcomes. A recent study in *BMC Systems Biology *provides an example of this, documenting a bimodal distribution of activated extracellular signal-regulated kinase in a population of cells exposed to epidermal growth factor and demonstrating that the observed bimodality of the response is induced purely by noise.

See research article: http://www.biomedcentral.com/1752-0509/6/109

## Commentary

Noise in biological systems is endemic and can contribute to biological phenotypes. For example, noise can affect fate determination in virus-infected cells by randomly switching between latency and reactivation, and it can also cause *Escherichia coli *to switch between competency and non-competency for DNA uptake. The origin of biological noise is attributed to randomness in biological reaction events, and leads to cell-to-cell variability in genetically identical cell populations. To understand noise-induced phenotypes, it is important to have the capability to observe behaviors at the single-cell level.

The paper by Birtwistle *et al*. [1] highlights an interesting issue with respect to noise in the mitogen-activated protein kinase (MAPK)/extracellular signal-regulated kinase (ERK) protein signaling pathway. This pathway receives environmental cues, such as changes in epidermal growth factor (EGF) that leads to an amplified signal in the form of activated ERK. The activation of ERK in turn results in diverse cellular responses, such as proliferation, differentiation, and apoptosis. In their study, flow cytometry was used to collect data in the form of fluorescence signals emitted from individual single cells. The signals indicated the activated ERK levels and showed a bimodal histogram in the cell population, which might otherwise have been obscured without adopting single-cell-level measurement techniques.

Bimodality can be the result of positive feedback mechanisms resulting in bistablity, a state very much like a light toggle switch. Such systems have been found in a number of natural systems so it was an obvious choice for Birtwistle *et al*. to investigate this possibility. However, although the ERK activation pathway has been found to exhibit bistability in some types of cell, it does not in others, and the experimental observations made by Birtwistle *el al*. in their cell line were not compatible with the mechanisms that used positive feedback. Closer investigation reveals something completely different and novel.

The observed bimodal distributions [1] were shown by computer modeling to be induced purely by noise without being related to positive feedback. There have been many theoretical studies to understand the mechanisms for such noise-induced bimodality. Many of these are related to the close interplay between nonlinearities in the system and noise in biological signals. The signal noise can be processed via a nonlinear input-output response, causing sufficient signal distortion to transform unimodal signal distributions into bimodal ones [2]. In the case of the signaling pathway studied by Birtwistle *et al*., the EGF signal is transmitted across the membrane to generate activated Ras, and the Ras signal, considered unimodal in the cell line used, activates the MAPK/ERK cascade after passing a threshold. The existence of this threshold means that the system is not purely linear, and signal distortion due to the system nonlinearity can explain the bimodality that emerges. This provides a first *in vivo *example in protein signaling networks that shows noise-induced bimodality due to its inherent nonlinear signal processing without positive feedback.

**Figure 1:**
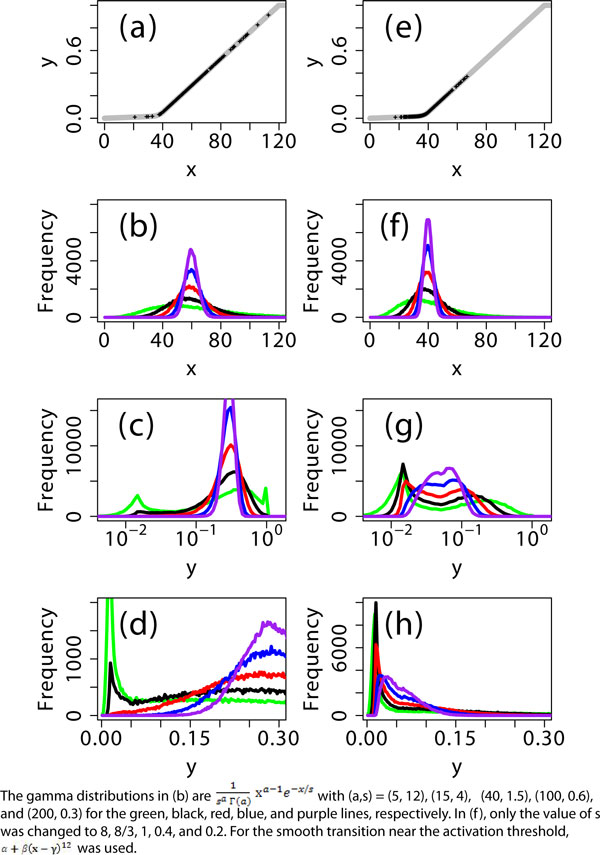
**Bimodal distribution in an output signal y due to cell-to-cell variability in an input signal x**. **(a) **The input-output response curve is piecewise linear with a smooth junction at the activation threshold (between × = 30 and 40; black dots correspond to the case of the 'black' line in (b)). **(b) **The input signal × was assumed to satisfy the Gamma distribution. **(c) **Bimodal and even trimodal distributions in the output signal y appear as the cell-to-cell variability in × increases while its mean value is fixed. The distributions or histograms were obtained after transforming × to log(x). **(d) **Some of the bimodal distributions in (c) still appear in a linear scale. **(e-h) **A smaller mean value of × was used. Bimodal distributions, observed at the log-scale, were highly suppressed or disappeared in a linear scale. The sample size for the distributions was 10^5^.

To understand the mechanism of bimodality observed by Birtwistle *et al*. [1], we will illustrate graded input-output response curves of the type that share their observed typical response patterns. These are piecewise-linear with a smooth connection at the activation threshold, as shown in Figure 1a. First, the response curve was assumed to be identical among all cells, that is, without cell-to-cell variability in the curve (Figure 1a). The input × was considered to be a certain type of a random number, satisfying a so-called Gamma distribution (Figure 1b). This distribution has been observed *in vivo *for *E. coli*, budding yeast, and mouse embryonic stem cells. It has been shown to appear when translation events occur in a bursting manner when a short-lived mRNA is transcribed [3]. When the distribution of × is sufficiently narrow, the response signal y can be shown to satisfy a unimodal distribution, but as the distribution of × gets wider, bimodal (black line in Figure 1c) and even trimodal (green line) distributions of y can appear. This multimodal distribution in y is caused by the fact that the threshold activation in the response curve distorts the distribution of the input signal [2]. This non-linear signal processing is the fundamental mechanism that caused the bimodality observed by Birtwistle *et al*.

Careful attention is, however, needed when computing distributions, more precisely histograms, depending on the choice of the scale of the x-axis. As in the case of flow cytometry, a signal is often visualized in the log scale to show the broad ranges of the signal values. This leads researchers to use the histogram of log-transformed signal values. One pitfall of this procedure is that it is possible to generate transformation artifacts. One example is shown in Figure 1, where a unimodal (or very weakly bimodal) distribution in a linear scale (Figure 1h) becomes a bimodal distribution in a log scale (Figure 1g). This is because the log-scale representation causes larger values of × to be compressed more visually, so that a greater number of samples will be taken at larger × values and fewer at smaller × values (Figure 1c,g). Therefore, flow cytometry data may need to be treated carefully. Birtwistle *et al*. represented their data in a log scale using Kaplan-Meier empirical cumulative distribution functions, which may reduce the problem. Although this method can visually amplify the bimodal distribution as the conventional log scale representation does, it was confirmed via personal communication that the bimodality robustly appears in the wide range of input doses (0.5 nM and 1 nM EGF in Figure 1B in Birtwistle *et al*. [1]) and the transformation artifact does not eliminate the bimodality except for the case of 0.1 nM EGF in the same figure.

**Figure 2:**
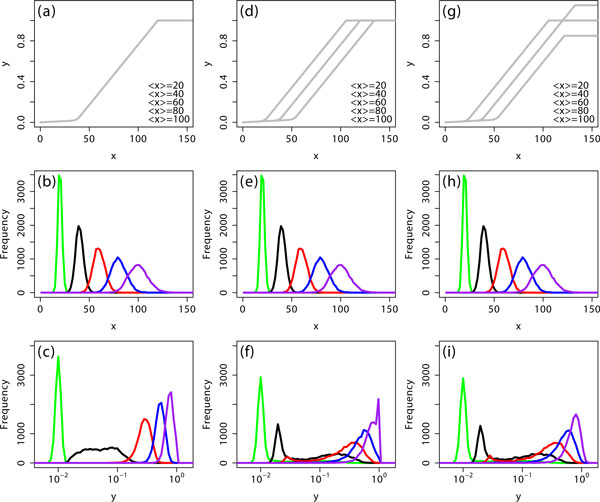
**Variability in the activation threshold enhances bimodality in the output signal y**. **(a-c) **A fixed input-output response curve was considered. **(d-f) **Variability in the activation threshold was introduced. **(g-i) **Additional variability in the saturation level of the response curve was considered. The simulation details are exactly the same except that the sample size was 10^4 ^and, for the Gamma distribution, the value of *a *was 100 (refer to Figure 1 legend). The variability in the activation threshold and the saturation level was generated from normal distributions.

Second, we consider cell-to-cell variability in the activation threshold. Figure 2a-c shows that unimodal distributions were obtained in a log-scale of x. For the same distributions in x, we introduced cell-to-cell variability in the activation threshold following a normal distribution (Figure 2d), resulting in bimodal distributions in y (Figure 2f). When shown in a linear scale, some bimodal distributions still persisted (for example, the red line in Figure 2f; graph not shown).

From these simulations, we can conclude that the variability in the input signal and the activation threshold both individually enhanced bimodality in the output signal. This is because the value of y is determined by the distance between the value of × and the activation threshold. Thus, the variability in the threshold has the same effect as the input variability by changing the distance between the value of × and the threshold. What happens if variability appears in both × and the threshold simultaneously? If the input signal and the threshold can be assumed to fluctuate independently due to the fact that they can be processed through sufficiently different biological systems, then the presence of the variability in both will enhance the bimodality further.

Finally, we can consider the variability in the saturation levels of the response curves. This variability smoothed the sharp peak appearing at the saturation level (y = 1) for the case that the saturation level was fixed (Figure 2f). Thus, the variability in the saturation level did not have any significant effect on bimodality.

The lesson from this work, and one that we see more and more often, is that the interaction of noise and the underlying deterministic dynamics can result in non-intuitive behavior. We are only beginning to understand how noise is exploited by nature [4] and furthermore by system designers like synthetic biologists [5], but the influence of noise is likely to be subtle and counter-intuitive to our normal deterministic view of the world.
